# Low tortoise abundances in pine forest plantations in forest-shrubland transition areas

**DOI:** 10.1371/journal.pone.0173485

**Published:** 2017-03-08

**Authors:** Roberto C. Rodríguez-Caro, Cornelia S. Oedekoven, Eva Graciá, José D. Anadón, Stephen T. Buckland, Miguel A. Esteve-Selma, Julia Martinez, Andrés Giménez

**Affiliations:** 1 Área de Ecología, Departamento de Biología Aplicada, Universidad Miguel Hernández, Av/ Universidad, Elche, Spain; 2 Centre for Research into Ecological and Environmental Modeling, University of St Andrews, The Observatory, Buchanan Gardens, St. Andrews, United Kingdom; 3 Department of Biology, Queens College, City University of New York, New York, New York, United States of America; 4 Departamento de Ecología e Hidrología, Área de Ecología, Universidad de Murcia, Murcia, Spain; 5 Fundación Nueva Cultura del Agua, Zaragoza, Spain; University of South Carolina, UNITED STATES

## Abstract

In the transition between Mediterranean forest and the arid subtropical shrublands of the southeastern Iberian Peninsula, humans have transformed habitat since ancient times. Understanding the role of the original mosaic landscapes in wildlife species and the effects of the current changes as pine forest plantations, performed even outside the forest ecological boundaries, are important conservation issues. We studied variation in the density of the endangered spur-thighed tortoise (*Testudo graeca*) in three areas that include the four most common land types within the species’ range (pine forests, natural shrubs, dryland crop fields, and abandoned crop fields). Tortoise densities were estimated using a two-stage modeling approach with line transect distance sampling. Densities in dryland crop fields, abandoned crop fields and natural shrubs were higher (>6 individuals/ha) than in pine forests (1.25 individuals/ha). We also found large variation in density in the pine forests. Recent pine plantations showed higher densities than mature pine forests where shrub and herbaceous cover was taller and thicker. We hypothesize that mature pine forest might constrain tortoise activity by acting as partial barriers to movements. This issue is relevant for management purposes given that large areas in the tortoise’s range have recently been converted to pine plantations.

## Introduction

Ecotones are areas with relatively sharp environmental gradients that possess unique natural attributes [1–4]. The southeastern Iberian Peninsula constitutes a biogeographic ecotone between the Mediterranean and arid subtropical biomes, and is the distribution limit for about 350 plant species [5, 6]. There is a progressive change from Mediterranean Forest, with *Pinus halepensis* as a dominant species, to the arid subtropical shrublands. Agriculture, fires and overgrazing have affected this ecotone since the Neolithic [7], leading to the existence of semi-natural or human landscapes characterized by habitat patches at different successional stages. These landscapes host a great biodiversity [8] and thus are key to conservation policies [9, 10]. In the last decades, however, these landscapes have suffered drastic changes due to crop abandonment, intensive agriculture, urbanization and reforestation [11].

Here we assess the effects of pine forests on a wildlife species in the forest-shrubland transition of the southeastern Iberian Peninsula. Reforestations in the Mediterranean have been based mostly on pine plantations to prevent erosion, floods, desertification, to assist or accelerate ecological succession from shrubs to original forest [12] and recently to mitigate anthropogenic carbon dioxide emissions [13]. In the Iberian Peninsula’s Murcia province, approximately 94,000 hectares (8% of the province) have been reforested in the last 110 years [14, 15]. Since the sixties, reforestations have been done mainly in the ecotone between Mediterranean forests and the arid subtropical shrublands (i.e., in areas with precipitation values close to the precipitation limit of the forests) [14, 16]. In this ecotone, most reforestations with *P*. *halepensis* outside the forest’s ecological boundaries (i.e., an afforestation) have frequently failed because the trees were in poor physiological condition, had poor growth, and had a high susceptibility to disease [17, 14, 15]. Pine plantations also have substantial negative consequences on other components of biodiversity, including a species-poor shrub understory [18]. Negative effects have also been described for birds [12, 19], insects [20, 21] and mammals [22]. However, studies addressing pine plantation effects on the herpetofauna are scarce (but see [23]).

The spur-thighed tortoise (*Testudo graeca*) is an endangered terrestrial tortoise inhabiting the ecotone between Mediterranean and arid subtropical areas [24]. The main threat to the species is habitat degradation, loss and fragmentation [25]. Within the distribution range of *T*. *graeca*, approximately 14% of available habitat has been the object of reforestation [14]. The main requirements of the species are sunny places for basking and shelters for protection from extreme temperatures and predators [9]. These two requirements are strongly dependent on the vegetation canopy which, in turn, could be affected by reforestation, so it is appropriate to evaluate *T*. *graeca* use of the landscape in the southeastern Iberian Peninsula [9]. In addition, *T*. *graeca*’s low mobility and dispersal abilities increase its vulnerability to local extinctions, which may be exacerbated by the impact of local changes in habitat quality [26–28].

The aim of this work is i) to understand the role of the different land-use types on the density of *T*. *graeca* and, in particular, ii) to study whether pine forests affect *T*. *graeca* population density. Density of tortoises have been widely estimated using line transect distance sampling methods [29–31]. For our study, we first used a two-stage modeling approach for distance sampling data [32] to estimate tortoise densities at the landscape scale in the principal habitat patches within the species’ range (i.e., shrubs, dry crops, pine forest and abandoned crops). For the second step, we described the vegetation cover of the habitat types at the microhabitat scale.

## Materials and methods

### Ethics statement

Permits for the field work and animal handling were provided by “La Delegación General de Medio Natural de la Comunidad Autónoma de la Región de Murcia” (AUT/ET/UND/48/2010).

### Study area and data collection

**Fig 1:**
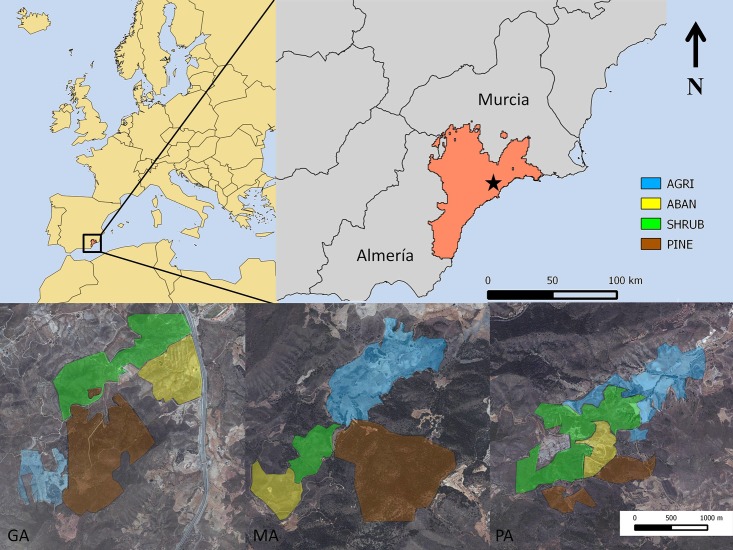
Location of the study sites in the southeastern Iberian Peninsula and distribution of habitat types within the field sites. The transects were performed in GA (Galera), MA (Madroñales) and PA (Palomera). Down panels are orthophotos of the PNOA (National Aerial Orthophotography Plan) provided by the Centro Nacional de Información Geográfica (http://centrodedescargas.cnig.es/CentroDescargas/index.jsp).

We estimated the abundance of *T*. *graeca* in different habitat types in the southeastern Iberian Peninsula. We chose three sample sites within the species’ range (Fig 1): Galera (GA), Madroñales (MA) and Palomera (PA). Each site was surveyed three times during spring 2012. The three sites each contain the four most representative land uses or habitat patches within the species’ distribution in the southeastern Iberian Peninsula [9, 33]. The four habitat patches were defined by their dominant land use or type of forest: pine forest (PINE, dominated by the Aleppo pine *P*. *halepensis*, one of the pine forests, GA, is the result of reforestation programs from the early 1980s); natural shrubs (NAT, with several species like *Anthillis cytisoides*, *Artemisia herba-alba*, *Rosmarinum officinalis*); dryland, tree-based crop fields (AGRI, such as olive *Olea euroapea* or almond tree *Prunus dulcis*); and abandoned crop fields (ABAND, where agricultural land has been abandoned and overgrown by bushes). The sampling effort was similar in each patch and each site; we covered around 0.33±0.19 km2 in area on each survey.

We surveyed tortoises using line transect distance sampling method [34], in spring (April and May, 2012), as this is the most important period of activity for the tortoises in this area [35]. Surveys were performed under adequate weather conditions (i.e. not during rain or temperatures below 18°C). Tortoises were detected visually by surveying along predefined lines through each of the four habitat types (PINE, NAT, AGRI and ABAND). We determined the sex of the tortoise according to secondary sexual characteristics [36], young individuals with no sex associated were classified as a subadults. Transect lines, around 1521±160 m long and straight, were randomly placed among habitat patches, covering the whole study area. A group of eight observers executed the surveys, walking simultaneously in four pairs, one pair worked in each habitat. Each site was surveyed in three consecutive days and observer pairs rotated between transect to avoid bias. For each tortoise detection, observers recorded the perpendicular distance from the tortoise to the transect line.

### Distance sampling analysis

For distance sampling calculations [34], we assumed detectability decreased as a function of perpendicular distance from the line and used the observed distances to model the detection function. In our study, we could not assume that the probability of detecting a tortoise on the line was certain, because the surface activity of tortoises has a strong seasonal pattern. For this purpose, we multiplied the data from a previously conducted radiotelemetry study to obtain an estimate of the percentage of surface-active tortoises during the monthly samples (April = 52.9%; May = 48.5%; using the percent of active tortoises from the total of tortoises with radio transmitters, [35]).

We adopted a two-stage modeling strategy to evaluate the relationship between tortoise densities and habitat [32, 37]. In the first stage, we fitted a detection function to the distance data using Distance 6.0 [38]. Upon preliminary inspection of the fit, we removed perpendicular distances beyond 4.25m (truncation distance, hereinafter ω) where detection probabilities generally fell to 0.1 or lower [34]. Due to the observers’ tendencies to round distances, we grouped the detections into five distance intervals (cutpoints: 0, 0.75, 1.5, 2.25, 3.25, 4.25 m; [34]). We evaluated the fit of the half-normal (HN) and hazard rate (HR) key functions with and without cosine series adjustments and explored covariate distance sampling techniques (MCDS) to model heterogeneity in detection probabilities. Covariates included were habitat patch (PINE, NAT, AGRI and ABAND), site (GA, MA and PA) and stage (ADULTS and SUBADULTS). Akaike’s Information Criterion (AIC, [39]) was used for model selection.

The best fitting detection model was then used to estimate the effective area that we included in the second-stage count model as an offset to account for imperfect detection within the surveyed strip (adjusting these counts using the data of a radiotelemetry study, [35]). In the second stage, we related adjusted counts to the covariates that may influence tortoise densities. Here, we used generalized linear models (GLM) with a log-link and a quasipoisson error structure. We also included habitat patch (hereafter, patch), site and the interaction of both as independent variables, since tortoise densities may vary among the sites or habitat patches. As in the first stage, we compared candidate models and selected the best model based on minimum AIC [40]. We used MuMIn package in R-project (R Core Team, 2013) to calculate a modification of Akaike's Information Criterion for overdispersed count data (QuasiAIC, QAIC).

### Vegetation structure analyses at microhabitat scale

Microhabitat structure was surveyed randomly around each transect (at 15-minute intervals), and at the location of each detected tortoise, in plots of diameter 3m. The cover of herbaceous vegetation, tree, shrub, perennial grasses and bare soil were noted visually and recorded for each plot (Table 1).

**Table 1:** Vegetation and soil categories assessed in the microhabitat analysis. Seven coverage categories were used for each variable: 1 = 0–1%; 2 = 2–10%; 3 = 11–33%; 4 = 34–50%; 5 = 51–66%; 6 = 67–90%; 7 = 91–100%.

Variable	Description
TREE	Coverage of trees
TALL	Coverage of tall shrub (> 150 cm height)
MEDIUM	Coverage of medium-sized shrub (between 50 cm– 150 cm height)
SMALL	Coverage of small shrub (< 50 cm height)
GRASSES	Coverage of perennial grasses
HERB	Coverage of herbaceous vegetation
BARE SOIL	Coverage of bare soil

We characterized the four habitat patches according to the vegetation structure. Discriminant function analysis (DFA, SPSS statistics 21) was performed to identify the variables that influence the differences between patches at microhabitat scale (differences in vegetation coverage in plots of diameter 3 m between patches). When we found differences in tortoise density in the same habitat patch among the three sites, we used DFA to explore differences in vegetation structure between these sites.

## Results

### Density model

We detected 251 tortoises but, after truncation at ω, we included 192 individuals in our analyses (S1 Table). The detection model with stage as a covariate provided the best fit (Table 2). Average detection probabilities were lower in subadults than adults. The detection probabilities and 95% confidence intervals were 0.629 (0.529–0.747) for adults and 0.315 (0.164–0.604) for subadults.

**Table 2:** Detection function models (first stage) for tortoises. The variable sites (GA, PA, MA), patches (AGRI, ABAND, NAT, PINE) and stage (ADULTS, SUBADULTS) were used in the analysis.

Key function^a^	Covariates	*K*	AIC
HN	Stage^b^	2	574.23
HR	Stage^b^	3	575.30
HN	Patch^b^	4	583.11
HN		1	583.43
HR		2	584.67
HR	Patch^b^	5	586.17
HN	Site^b^	3	586.85
HR	Site^b^	4	588.55

K = Number of parameters in the model

^a.^ Key function models—hazard rate (HR) and half normal (HN)

^b.^ Factor covariates

**Fig 2:**
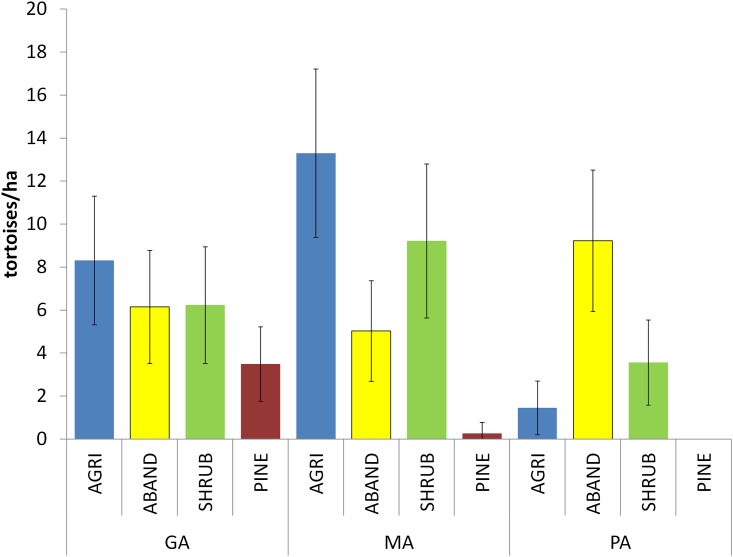
Density of tortoises (tortoises/ha ± confidence intervals) by site and patch type, derived from the best fitting quasipoisson count model (i.e., habitat x site).

The regression model (second stage) indicated that tortoise abundance was affected by the interaction between patch and site (Table 3). Tortoise densities were greater in dryland, tree-based crop fields, abandoned crop fields and natural shrub patches (d_mean_ = 7.97, 6.93 and 6.34 tortoises/ha, respectively) than in pine forest (d_mean_ = 1.25 tortoises/ha). We also detected tortoise density differences between sites in tree-based agriculture and pine forests (GLM results in S2 Table). Densities in tree-based agriculture were lower in PA than in GA and MA (P.value < 0.001). Density in pine forests was higher in GA than in PA (no tortoises were found there) and MA (P.value = 0.029; Fig 2).

**Table 3:** Factors affecting the density of tortoises (second stage). Results of generalized linear models (GLM) with a log-link and a quasipoisson error structure. QuasiAIC was used for model selection. Degrees of freedom (df) and percentage of explained deviance (Dev) are also shown.

Model	QAIC	ΔQAIC	df	Dev
Patch x site	207.7	0.0	12	58.5
Patch + site	259.2	51.5	6	30.8
Patch	270.3	62.6	4	24.4
Site	312.8	105.1	3	5.7
Intercept only	322.4	114.7	1	0

### Vegetation structure analyses at microhabitat scale

**Fig 3:**
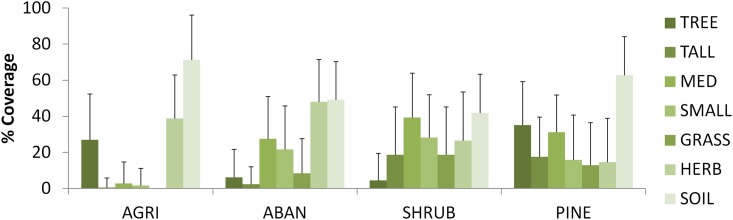
Mean cover of each environmental variable significant in the DFA per patch. Pine forest (PINE), natural shrubs (NAT), dryland tree-based crop fields (AGRI) and abandoned crop fields (ABAND). See Table 1 for categories of vegetation cover (TREE, TALL, etc.). AGRI = 216, ABAND = 155, NAT = 158 and PINE = 127 samples.

We recorded 656 vegetation samples (we recorded 241 vegetation samples associated with tortoises encounters, and we randomly recorded 415 vegetation samples in each transect). At microhabitat scale, the DFA derived three discriminant functions. The first and second functions explained 97.7% of the variation. The pooled within-groups correlations to the first function are related positively with the cover of medium-sized and small shrubs and negatively to the coverage of bare soil. This function can be interpreted as a gradient from bare soil to dense shrub coverage. The second function indicated the influence of tall and thick vegetation because the coverage of trees and tall shrubs contributed positively. Using just the first two functions, we can describe the different patches (See S1 Fig). Dryland crop fields were described as areas with high values of bare soil and trees (Fig 3). Abandoned crop fields were characterized by a cover of shrubs without trees or tall shrub, and high cover of herbaceous vegetation. There was a high cover of plants in natural shrublands, in particular of medium and small-sized shrubs. In pine forest, trees and tall shrubs were dominant.

**Fig 4:**
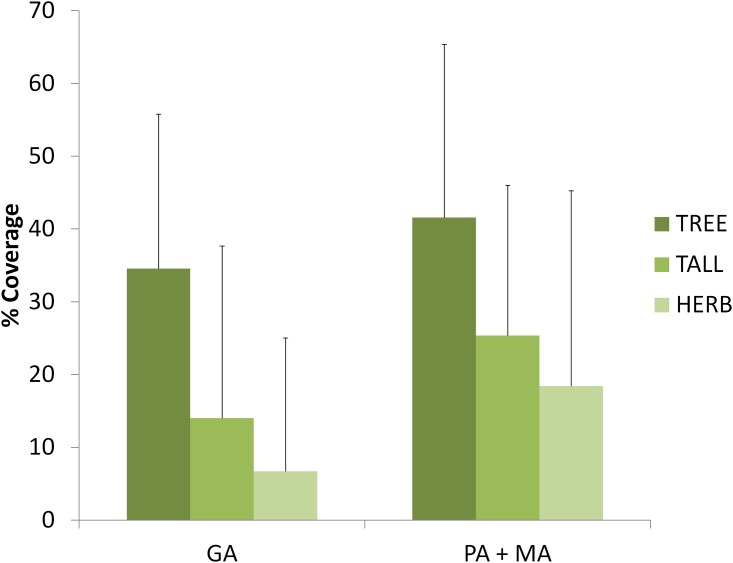
Comparison of microhabitat characteristics of pine forests between patches with high and low density of tortoises. MA and PA show lower density than site GA and thus are merged. See Table 1 for categories of vegetation cover.

Since we found site differences in tortoise densities in pine forest, we compared the vegetation coverage of pine forest in two groups, with high density of tortoises (GA) and low density of tortoises (MA and PA). The DFA created just one function, which was significant (χ^2^ = 56.853, df = 4, P < 0.001). This function can be interpreted as a gradient of vegetation cover (with the discriminating variables: TALL = 0.704, HERB = 0.332, TREE = 0.144, see Table 1 for categories of vegetation cover). Both kinds of pine forest were characterized in different positions of the discriminant function, GA forest was negatively related to the function (-0.981) and, MA and PA forests were positively related (0.818). According to these analyses, the mean amount of vegetation coverage (TALL, HERB and TREE) was higher in MA and PA than in GA (Fig 4a).

We also tested the differences in AGRI between the low density site (PA) and the other two sites (GA and MA). We did not find differences in the vegetation structure of AGRI patches among locations using DFA (P > 0.05).

## Discussion

Two-stage modelling was a good approach to disentangle the factors affecting detectability and density of tortoises. As in previous works, we found that detectability was affected by individuals’ age class: juveniles versus adults [29, 30, 41]. On the other hand, density was also affected by habitat patches and sites. According to abundance classes defined in a previous study [42], the density estimates of *T*. *graeca* found in this work for the three sites (GA = 6.35; MA = 6.95; PA = 3.56 ind/ha) showed high values within the southeastern Iberian Peninsula,. As has been shown in previous studies, reptiles prefer landscapes with mosaic structure for thermoregulation because they contain basking places near shadier areas where animals can cool and find shelter [9, 43]. In our study, differences in the density of tortoises were found at two scales. Firstly, at a patch scale, the lowest tortoise densities were found in pine forest. Trees and dense vegetation cover in pine forest patches may explain the low tortoise abundance, since open areas are needed to fulfil the thermoregulation requirements of tortoises [44]. Previous studies [9, 33] showed that there is a positive relationship between the presence of *T*. *graeca* and shrubland cover when the shrubland cover is 75% or less. Those authors also showed that *T*. *graeca* prefer re-colonize shrublands and cropland rather than more complex mature shrublands. Our results accord with previous works showing that densely forested areas such as pine forest, have a negative effect on reptiles [45, 46] and that tortoises prefer open habitats [47, 48], such as dryland or abandoned crops fields and natural shrub. Secondly, at the microhabitat scale, differences in tree-based agriculture patches were not so evident. Only the cover of bare soil seemed to differ between sites (mean cover of bare soil in AGRI for PA was 82.05% and 63.70% in GA and MA). However, this relation could have resulted from excessive ploughing in the tree-based agriculture areas of PA (authors, pers. obs.). The high percentage of bare soil can influence other mechanism as predation by the absence of shelter.

**Fig 5:**
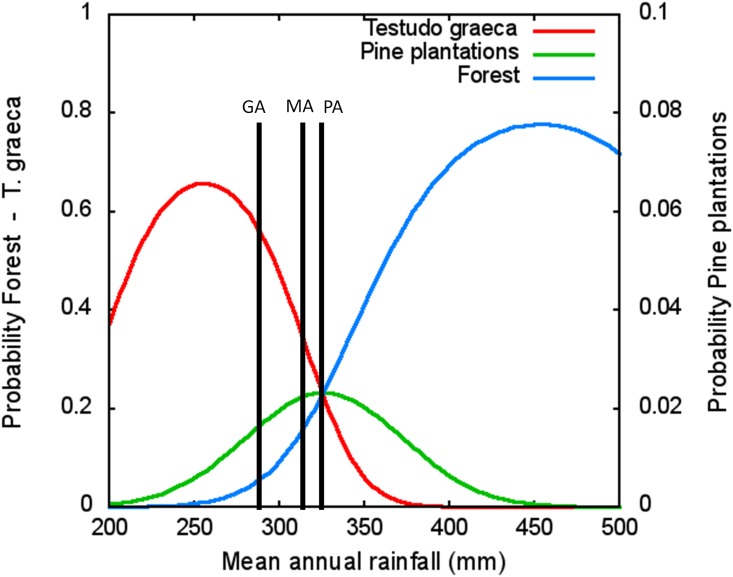
Probability of presence of natural forests and pine plantations in southeastern Iberian Peninsula [14, 16] and, probability of presence of *T*. *graeca* [33] as a function of mean annual rainfall, according to statiscally significant distribution models. GA, MA and PA sites were represented according their annual rainfall.

In relation to pine forest, there were also important differences at the microhabitat scale. Forests of diverse origin (i.e. natural or as a result of reforestations) are common throughout the range of *T*. *graeca* in the southeastern Iberian Peninsula. Pine forest of GA is the result of reforestation programs from the early 1980s and, we found in our results that microhabitat characteristics in GA were different than in the other two sites (PA and MA). As we found in the discriminant analysis, early reforested pine forest of GA showed lower shrub coverage compared to other sites. Besides the age of the reforestation, the structure of the vegetation between the pine plantations in GA and the pine forests in the other two sites may also be explained by the climate differences among the sites. GA is slightly dryer than the other two study sites (290 mm vs 310–320 mm of average annual rainfall). Because our study sites were located in a forest-shrubland transition area, this small difference in rainfall produces notable differences in the kind of forest and in the viability of pine plantations [14, 15]. According to regional climatic models (Fig 5, [14]), the probability of occurrence of pine forest is 5% in GA and ranges from 10 to 20% in the other two sites. Thus, pine plantations in GA are younger and pines have worse conditions than the other two study sites. These differences lead to lower canopy cover values, more open areas and a better habitat quality for thermoregulation purposes of *T*. *graeca* (i.e. [9, 33]).

From a conservation perspective, our results suggests that pine forest might act as a partial barrier for populations at a landscape scale. The impact of pine forest plantations on *T*. *graeca* distribution areas has the potential to be higher at higher precipitation levels due to the dense forest canopies. Forest conservation policies mainly aim to prevent deforestation [49], but there is an increasing awareness that an excess of forest cover may also be problematic, especially in human-altered landscapes in temperate regions [50], and particularly for some reptile species [45, 46]. Pine forest plantations are a low-quality habitat for different species of reptiles [51, 52]. Recent studies pointed the importance of consider thermal requirements of forest plantations for richness and abundance of reptiles [53]. Current conservation polices are not focused on preserving the mosaic structure of the landscape. However, maintaining the heterogeneity of these mosaic traditional landscapes is key to prevent the loss of biodiversity [10]. In this sense, results from this study should be considered in nature conservation plans, specifically in the management of the Natura-2000 network in the southeastern Iberian Peninsula (Directive 92/43/EEC, http://ec.europa.eu/environment/nature/legislation/habitatsdirective/index_en.htm). New policies should match the need of restoring forest in those areas where they have been historically replaced by shrubs, and the conservation of species like the spur-thighed tortoise that is linked to the current heterogeneous landscapes with patches of open habitats.

## Supporting information

S1 Table Distribution of observation between sites and habitat patch. Tortoises found during the surveys.
(DOCX)

S2 Table Parameter estimates from the best approximating, generalized linear model with Standard Error (SE). We show the results of the GLM with model site-patch interaction. We found significant differences in the interaction between PA-AGRI and MA-PINE, in both the density is lower than in the other sites.
(DOCX)

S1 Fig Results of the DFA analysis. The first function (the gradient from bare soil to dense shrub coverage) is showed in the X axis, the second function (the influence of tall and thick vegetation) in the Y axis. The 656 vegetation samples are showed in different colors according habitat type and, finally, the centroids for each habitat are also showed.
(DOCX)

S1 Dataset Surveys’ data. (RAR)
