# Supplementary material for: Low tortoise abundances in pine forest plantations in forest-shrubland transition areas
Source: PLoS One. 2017 Mar 8;12(3):e0173485. doi: 10.1371/journal.pone.0173485 (PMC5342264; doi:10.1371/journal.pone.0173485)
Supplement: S1 Table — Tortoises found during the surveys. (DOCX) [file pone.0173485.s001.docx]

**S1 Table. Distribution of observation between sites and habitat patch**

| **Site** | **AGRI** | **ABAND** | **SHRUB** | **PINE** | **TOTAL** |
| --- | --- | --- | --- | --- | --- |
| Galera | 34 | 23 | 24 | 20 | 101 |
| Madroñales | 67 | 20 | 17 | 1 | 105 |
| Palomera | 5 | 32 | 8 |  | 45 |
| **Total** | **106** | **75** | **49** | **21** | **251** |
