# Supplementary material for: Low tortoise abundances in pine forest plantations in forest-shrubland transition areas
Source: PLoS One. 2017 Mar 8;12(3):e0173485. doi: 10.1371/journal.pone.0173485 (PMC5342264; doi:10.1371/journal.pone.0173485)
Supplement: S2 Table — We show the results of the GLM with model site-patch interaction. We found significant differences in the interaction between PA-AGRI and MA-PINE, in both the density is lower than in the other sites. (DOCX) [file pone.0173485.s002.docx]

**S2 Table. Parameter estimates from the best approximating, generalized linear model with standard error (SE)**. Interaction effects between variables (*) are also shown.

|  | Estimate | SE | p.value |
| --- | --- | --- | --- |
| Intercept | -7.3319 | 0.21816 | **<0.001** |
| MA | -0.3026 | 0.3323 | n.s. |
| PA | 0.3895 | 0.2854 | n.s. |
| AGRI | 0.337 | 0.2834 | n.s. |
| PINE | -0.4696 | 0.3255 | n.s. |
| NAT | -0.4096 | 0.3513 | n.s. |
| MA*AGRI | 0.7485 | 0.4067 | 0.069 |
| PA*AGRI | -2.2318 | 0.5647 | **<0.001** |
| MA*PINE | -2.4534 | 1.1094 | **0.029** |
| PA*PINE^1^ | NA | NA | **NA** |
| MA*NAT | 0.8163 | 0.4893 | n.s. |
| PA*NAT | -0.7581 | 0.5084 | n.s. |

^1^ No tortoises were found in the pine forest (PINE) of Palomera (PA), so we excluded PA’s pine forest from the analysis.
