# Supplementary material for: Low tortoise abundances in pine forest plantations in forest-shrubland transition areas
Source: PLoS One. 2017 Mar 8;12(3):e0173485. doi: 10.1371/journal.pone.0173485 (PMC5342264; doi:10.1371/journal.pone.0173485)
Supplement: S1 Fig — The first function (the gradient from bare soil to dense shrub coverage) is showed in the X axis, the second function (the influence of tall and thick vegetation) in the Y axis. The 656 vegetation samples are showed in different colors according habitat type and, finally, the centroids for each habitat are also showed. (DOCX) [file pone.0173485.s003.docx]

**S1 Fig. Results of the DFA analysis.**


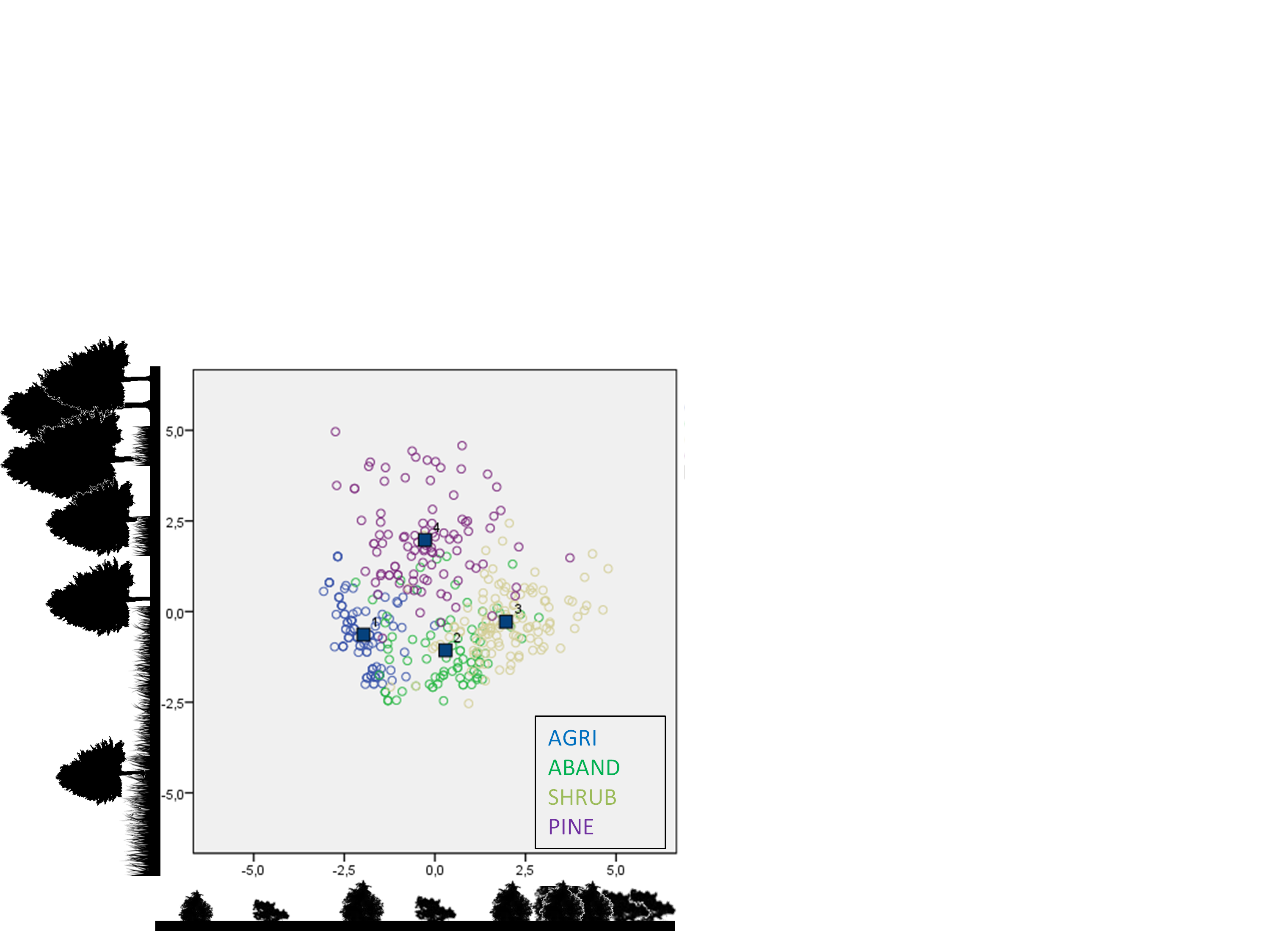


The first function (the gradient from bare soil to dense shrub coverage) is showed in the X axis, the second function (the influence of tall and thick vegetation) in the Y axis.
